# Complementary and alternative therapies for knee osteoarthritis: A protocol for systematic review and network meta-analysis

**DOI:** 10.1097/MD.0000000000023035

**Published:** 2020-10-30

**Authors:** Haiyang Yu, Haiyan Wang, Panju Cao, Tao Ma, Yongli Zhao, Feiyang Xie, Chuanjiang Yao, Xiaogang Zhang

**Affiliations:** a Clinical College of Traditional Chinese Medicine, Gansu University of Chinese Medicine; b Department of Orthopedics; c Department of Acupuncture and Moxibustion, Affiliated Hospital of Gansu University of Chinese Medicine, Lanzhou, Gansu Province, China; d College of Acupuncture and Tuina, Chengdu University of Traditional Chinese Medicine, Chengdu, Sichuan Province; e Department of Spine, Baoji Hospital of Traditional Chinese Medicine, Baoji, Shanxi Province, China.

**Keywords:** complementary and alternative therapies, knee osteoarthritis, protocol, systematic review

## Abstract

**Background:**

Knee osteoarthritis (KOA) is a degenerative disease, making a unique contribution to chronic pain, edema, and limited mobility of knee joint. This disease is an important factor affecting the quality of life of middle-aged and elderly people. Complementary and alternative medicine (CAM) therapies have been used clinically to treat KOA; however, the selection strategies of different CAM interventions in clinical practice are still uncertain, and the purpose of this study is to evaluate the efficacy and acceptability of different CAM therapies using systematic review and network meta-analysis.

**Methods:**

According to the strategy, the authors will retrieve a total of 7 electronic databases by October 2020, including PubMed, the Cochrane Library, EMbase, China National Knowledge Infrastructure, China Biological Medicine, Chongqing VIP, and Wan-fang databases After a series of screening, 2 researchers will use Aggregate Data Drug Information System and Stata software to analyze the data extracted from the randomized controlled trials of CAM therapies for the KOA. Finally, the evidence grade of the results will be evaluated.

**Results:**

This study will provide a reliable evidence for the selection of CAM therapies for KOA.

**Conclusion:**

The results of this study will provide references for evaluating the influence of different CAM therapies for KOA, and provide decision-making references for clinical research.

**Ethics and dissemination:**

This study does not require ethical approval. The results will be disseminated through a peer-reviewed publication.

**OSF registration number:**

DOI 10.17605/OSF.IO/GJMF4.

## 1 Introduction

Knee osteoarthritis (KOA) is one of the most common forms of arthritis, with pain and limited function as the main causes.^[1,2]^ Only in the U.S., reportedly 27 million people are affected by knee osteoarthritis with associated treatment costs of $185.5 billion per year.^[3]^ In China, 19.4% of the elderly population which is over 60 years old suffer from KOA.^[4,5]^ According to the Bulletin of the World Health Organization, osteoarthritis is predicted to be the fourth leading cause of disability by 2020.^[6]^ Patients often have symptoms such as pain and dysactivity, which limits the mobility of patients, causes psychosocial problems such as low self-confidence and depression, and decreases the quality of life.^[4,7,8]^

The main focus of the treatment has been to relieve pain, to restore function, and to slow the progression of the disease.^[9,10]^ The treatments have been classified as pharmacological, non-pharmacological, and surgical or combinations of these.^[11]^ For the non-surgical treatment of KOA, pharmacologic treatments were the most widely adopted. Anti-inflammatory drugs as well as non-opioid analgesics have been prescribed for the reduction of inflammation and pain.^[12]^ Although these have effectively reduced the inflammation and pain, they have led to undesirable side effects in long-term follow-up studies, such as digestive problems, heart failure, and renal impairment.^[13]^ However, potential side effects of long-term pharmacological therapy limited their use.^[14]^ Consequently, many individuals have turned their attention to some other treatments, such as complementary and alternative medicine (CAM).

CAM generally refers to techniques that are integrated with or substituted for traditional practices of western medicine.^[15]^ In recent years, with the continuous development of CAM research, it has achieved significant results in the treatment of knee osteoarthritis. At the same time, with its unique advantages such as multitarget therapeutic effects and low toxic and side effects, it has obtained a large number of patients and doctors.^[16–19]^ An increasing number of researchers are studying the use of CAM approaches for treating KOA, including acupuncture, moxibustion, Tuina, Chinese herbal medicines, yoga, Baduanjin, and Tai Chi.^[20–24]^

There are several CAM therapies for KOA and their efficacy has been assessed by some systematic reviews.^[25–30]^ However, there has been no network meta-analysis (NMA) of the differences between different CAM therapies for KOA. The aim of this study is to assess the efficacy and acceptability of different CAM therapies, and to provide a clinically useful reference of the comparative evidence that can be used to guide decisions about the treatment of KOA.

## 2 Methods

### 2.1 Protocol and registration

This protocol follows the Preferred Reporting Items for Systematic Reviews and Meta-Analyses Protocols (PRISMA-P) guidelines.^[31]^ The NMA protocol has been registered on Open Science Framework (OSF) platform (https://osf.io/9r3yq/), registration number: DOI 10.17605/OSF.IO/GJMF4.

### 2.2 Eligibility criteria

#### 2.2.1 Type of participants

Studies of adults (18 years of age and older) with osteoarthritis of the knee were eligible. All studies including patients with KOA diagnosed by any set of criteria were eligible for inclusion, such as Diagnostic and Statistical Manual of Mental Disorders (DSM-5), International Classification of Diseases (ICD-10), regardless of gender, ducational background, nationality, or outpatient therapy or inpatient therapy.

#### 2.2.2 Type of interventions and comparators

Complementary and alternative therapies for treating MOA include acupuncture, moxibustion, Chinese herbal medicines, yoga, baduanjin, tuina, and Tai Chi. These interventions can be used alone or in combination. Controlled interventions included control groups with no treatment, sham/placebo groups, or other conventional treatments.

#### 2.2.3 Type of outcomes

##### 2.2.3.1 Primary outcomes

Total score of the Western Ontario and McMaster Universities Osteoarthritis Index (WOMAC)^[32]^ includes pain, physical functional, or stiffness. The higher the global WOMAC score is, the worse the function of knees is.

##### 2.2.3.2 Secondary outcomes

The secondary outcomes will include the following:

1. Frequency and nature of adverse events.
2. Physical performance tests include: changes in gait kinematic measures, Short Physical Performance Battery (SPPB),^[33]^ Lower Extremity Functional Scale (LEFS),^[34]^ knee joint proprioception, Visual Analogue Scale (VAS),^[35]^ knee range of motion (ROM),^[36]^ Berg Balance Scale (BBS),^[37]^ and Timed “up and go” test.
3. Measurements to assess quality of life include: the Pittsburgh Sleep Quality of Index (PSQI)^[38]^ and short form health survey (SF-36).^[39]^

#### 2.2.4 Study design

This study is a systematic review and network meta-analysis of randomized controlled trials (RCTs) with CAM therapies on KOA. This research will include all relevant RCTs using CAM therapies for KOA and the first period in randomized cross-over trials, regardless of publication status. Quasi-RCTs, review documents, clinical experience, and case reports. Moreover, we will only search English and Chinese literature in this study. And we will remove the studies without comparable baselines and duplicate publications.

### 2.3 Literature retrieval strategy

Computer retrieval of published RCTs of complementary alternative therapy for KOA is conducted in PubMed, the Cochrane Library (issue 10, 2020), EMbase, China National Knowledge Infrastructure (CNKI), China Biological Medicine (CBM), Chongqing VIP, and Wan-fang databases. The time limit of document retrieval is from the establishment of each database to October 31, 2020. Using medical subject heading (MeSH) terms and key words to identify RCTs with the limitation of Chinese and English language. In addition, inclusive literature from the field and references from previous evaluations will be manually retrieved to find other potentially relevant articles. Chinese search terms mainly include: “knee osteoarthritis”; English search words include “knee osteoarthritis,” “KOA,” “acupuncture,” “moxibustion,” “yoga,” “tui na,” “baduanjin,” “Tai Chi,” etc. Taking PubMed as an example, the initial retrieval strategy is shown in Table 1 and will be adjusted according to the specific database.

**Table 1:**
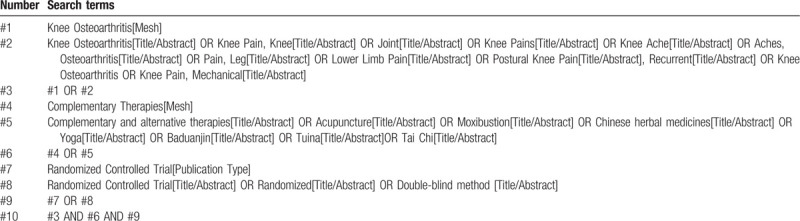
Search strategy of the PubMed.

### 2.4 Literature selection and data extraction

**Figure 1:**
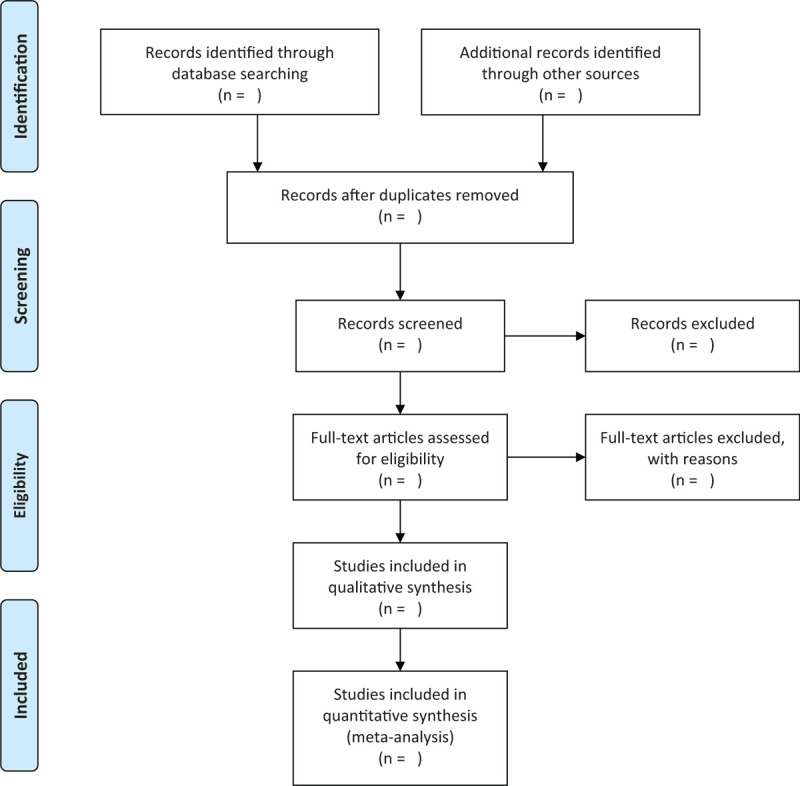
Flow chart of literature screening.

The study selection program will follow the Prisma guidelines, as shown in Fig. 1, Haiyang Yu and Haiyan Wang will independently screen literatures according to inclusion and exclusion criteria and cross-checked against:

1. Preliminary screening of the literature through Endnote software to remove duplicates;
2. By reading the title and preliminarily screening the abstract, exclude the literature that obviously does not meet the inclusion criteria;
3. Download and read the full text for rescreening.

At the end of the filtering, the extracted features are recorded using a pre-designed data table. These features include title, journal, author, publication year, country, sample size, gender, mean age, intervention, comparator, course of treatment, outcome measures, and follow-up time. If there is any disagreement, the third researcher Panju Cao will be asked to assist in the judgment. At the same time, the key factors of bias risk assessment are extracted.

### 2.5 Quality assessment

The quality of systematic review reflects the risk of bias or validity in its process and results, as well as the reliability of the included studies. The quality of the included studies will be assessed according to the Cochrane Reviewers’ Handbook. Two trained researchers Haiyang Yu and Panju Cao will independently evaluate the risk of bias of the included studies. If the results are disputed, they will be submitted to the corresponding author (Xiaogang Zhang) of this study for review and determination.

Cochrane Reviewers’ Handbook will be used to assess the risk of RCTs being included in NMA, including^[40]^:

(1) random sequence generation;
(2) allocation concealment;
(3) blinding of the subjects and researchers;
(4) blinding of outcome assessment;
(5) incomplete outcome data;
(6) selective reporting;
(7) other bias.

### 2.6 Data synthesis and statistical methods

#### 2.6.1 Network meta-analysis

This study uses Aggregate Data Drug Information System (ADDIS) 1.16.8 based on Bayesian framework for NMA.^[41]^ Odds ratios (ORs) or standardized mean differences (SMD) will be modeled using Markov chain Monte Carlo methods, both with 95% confidence intervals (CIs). Preset model parameters: 4 chains are used for simulation analysis, with an initial value of 2.5, a step size of 10,20,000 annealing times, and 50,000 simulation iterations. The network evidence plot will be generated according to different outcome. According to the results of the NMA, rank probability plot of various CAM therapies will be generated and sorted by dominance, with Rank1 being the optimal sort.

#### 2.6.2 Consistency assessments/statistical model selection

The Node-split model is used to check for consistency between direct and indirect evidence. If there is no statistical difference (*P* > .05) between direct comparison and indirect comparison, the consistency model is used, whereas the inconsistency model is used for analysis. If the consistency model is adopted, then the stability of the results is verified by the inconsistency model: when the inconsistency factors including 0, at the same time inconsistency standard deviation including 1 says the result of consistency model is more stable and reliable. At the same time, various analysis models are iterated with preset parameters, and the convergence of iteration effect is judged by potential scale reduced factor (PSRF). When the PSRF value is close to or equal to 1 (1 ≤ PSRF ≤ 1.05), the convergence is complete, the model has good stability, and the conclusion of analysis is reliable. If the PSRF value is not in this range, the iteration continues manually until the PSRF value reaches the range standard.

#### 2.6.3 Heterogeneity test

Before the combination of effect size, we will use Stata to assess available study and patient characteristics to ensure similarity and to investigate the potential effect of heterogeneity on effect estimates. When inter-study heterogeneity exists, the random effect model is used. For comparison of each pair, heterogeneity is assessed by the statistic *I*^2^ value. When *I*^2^ > 50%, it indicates that there is heterogeneity between studies, and the source of heterogeneity should be further searched. When *I*^2^ < 50%, inter-study heterogeneity is considered to be small or there is no obvious heterogeneity.

#### 2.6.4 Sensitivity analysis

If necessary, the sensitivity analysis will be used to assess the effect of each study on the random effects model. The sensitivity of the general combined effect of all outcome indicators is analyzed by the exclusion method. That is, each study is excluded, and the remaining studies will be re-analyzed to identify the stability of the results. If there is no qualitative change in the combined effect showed in the results, the results are stable.

#### 2.6.5 Subgroup analysis

If necessary, we will conduct a subgroup analysis of duration of treatment, age, the course of KOA, and research quality.

#### 2.6.6 Small sample effect/publication bias

If 10 or more studies are included in the NMA, a comparison-adjusted funnel plot is developed using Stata to evaluate the presence of small sample effects or publication bias in the intervention network. Descriptive analysis will be carried out through the symmetry of funnel plot. If the plot is asymmetric and there is no inverted funnel shape, it indicates that there may be publication bias. This may be related to the difficulty in the publication of the literature with negative results and the low quality of the inclusion methods.

#### 2.6.7 Dealing with missing data

If the required data is lost or incomplete, we will contact the corresponding author of the original document or the relevant email address of the first author. If there is no response, the record is excluded.

#### 2.6.8 Evaluating the quality of the evidence

To grade evidence quality and understand the current situation of evidence rating thereby analyzing possible problems, The Grading of Recommendations Assessment, Development and Evaluation (GRADE) instrument will be used to assess the quality of evidence in the NMA.^[42]^ Based on the risk of bias, inconsistency, imprecision, indirection, and publication bias, GRADE grades evidence quality into 4 levels: high, medium, low, and very low.

## 3 Discussions

KOA is a disabling joint disease with a high-prevalence among the middle-aged and old individuals. The pain, stiffness, and dysfunction of knees bring about poor sleep quality and negative moods involving depression, anxiety, and tension, which directly lower the quality of life. For cost issues and patient concerns regarding analgesic-related adverse effects causing by analgesic medications, there has been a growing number of studies on CAM therapies for KOA in the late years, compared with the current medication treatment for KOA, CAM therapies have special strengths, it may not carry the risks of side effects from pharmaceutical approaches.^[43]^

With an increasing amount of publications on CAM therapies for patients with KOA in recent years, we would like to figure out which has the relatively optimal effect and safety among those interventions. Given that systematic reviews with high quality can help provide best evidence in clinical practice, and a network meta-analysis can offer a ranking result based on comparative effectiveness, safety and costs, we conceive and design this study protocol. Thus, our study employed a NMA of all RCTs of CAM therapies for KOA, including acupuncture, moxibustion, Chinese herbal medicines, yoga, Baduanjin, Tai Chi, etc, to synthesize all this evidence and perform an integrated rank of available CAM treatments for KOA.

There are some potential limitations predictable in this study. First, a number of studies we included were of low quality. Few RCTs comparing interventions with controls were available, limiting the number of studies that could be included in the meta-analysis. In addition, due to the limitations of language ability, the authors only search for literature in English and Chinese, and may lead to the potential risk of ignoring essential literature.

## Author contributions

**Conceptualization:** Haiyang Yu, Haiyan Wang, Panju Cao

**Data curation:** Haiyang Yu, Haiyan Wang, Tao Ma

**Formal analysis:** Yongli Zhao, Feiyang Xie

**Funding acquisition:** Xiaogang Zhang.

**Methodology:** Haiyang Yu, Haiyan Wang, Panju Cao

**Project administration:** Haiyang Yu, Tao Ma, Chuanjiang Yao

**Writing – original draft**: Haiyang Yu, Haiyan Wang

**Writing – review & editing:** Xiaogang Zhang.

## References

[1] Ma VY Chan L Carruthers KJ Incidence, prevalence, costs, and impact on disability of common conditions requiring rehabilitation in the United States:stroke, spinal cord injury, traumatic brain injury, multiple sclerosis, osteoarthritis, rheumatoid arthritis, limb loss, and back pain. Arch Phys Med Rehabil 2014;95:986–95. 24462839 10.1016/j.apmr.2013.10.032 PMC4180670

[2] McAlindon TE Bannuru RR Sullivan MC OARSI guidelines for the non-surgical management of knee osteoarthritis. Osteoarthr Cartil 2014;22:363–88. 10.1016/j.joca.2014.01.003 24462672

[3] Nguyen US Zhang Y Zhu Y Increasing prevalence of knee pain and symptomatic knee osteoarthritis: survey and cohort data. Ann Intern Med 2011;155:725–32. 22147711 10.1059/0003-4819-155-11-201112060-00004 PMC3408027

[4] Xie Y Yu Y Wang JX Health-related quality of life and its influencing factors in Chinese with knee osteoarthritis. Qual Life Res 2020;29:2395–402. 32314125 10.1007/s11136-020-02502-9

[5] Zhang Y Jordan JM Epidemiology of osteoarthritis. Clin Geriatr Med 2010;26:355–69. 20699159 10.1016/j.cger.2010.03.001 PMC2920533

[6] Woolf AD Pflfleger B Burden of major musculoskeletal conditions. Bull World Health Organ 2003;81:646–56. 14710506 PMC2572542

[7] Riddle DL Kong X Fitzgerald GK Psychological health impact on 2- year changes in pain and function in persons with knee pain: data from the osteoarthritis Initiative. Osteoarthr Cartil 2011;19:1095–101. 10.1016/j.joca.2011.06.003 PMC3159740 21723400

[8] Vitaloni M Botto-van Bemden A Sciortino Contreras RM Global management of patients with knee osteoarthritis begins with quality of life assessment: a systematic review. BMC Musculoskelet Disord 2019;20:493. 31656197 10.1186/s12891-019-2895-3 PMC6815415

[9] Zhang W Moskowitz RW Nuki G OARSI recommendations for the management of hip and knee osteoarthritis, Part II: OARSI evidence-based, expert consensus guidelines. Osteoarthr Cartil 2008;16:137–62. 10.1016/j.joca.2007.12.013 18279766

[10] Schurman DJ Smith RL Osteoarthritis: current treatment and future prospects for surgical, medical, and biologic intervention. Clin Orthop Relat Res 2004;427:S183–9. 15480065

[11] Ye J Cai S Zhong W Effects of tai chi for patients with knee osteoarthritis: a systematic review. J Phys Ther Sci 2014;26:1133–7. 25140112 10.1589/jpts.26.1133 PMC4135213

[12] Shi X Yu W Wang T A comparison of the effects of electroacupuncture vs transcutaneous electrical nerve stimulation for pain control in knee osteoarthritis: a protocol for network meta-analysis of randomized controlled trials. Medicine 2019;98:e16265. 31305408 10.1097/MD.0000000000016265 PMC6641830

[13] Zhang H Liu T Li F A random, case–control study on the efficacy and safety of Wish Bitong Xifang fumigation for mild and moderate knee osteoarthritis patients. Int J Rheum Dis 2015;18:502–7. 24251557 10.1111/1756-185X.12165

[14] Montalescot G Sechtem U Task Force Members. 2013 ESC guidelines on the management of stable coronary artery disease: the Task Force on the management of stable coronary artery disease of the European Society of Cardiology. Eur Heart J 2013;34:2949–3003. [published correction appears in Eur Heart J. 2014 Sep 1;35(33):2260–1]. 23996286 10.1093/eurheartj/eht296

[15] Institute of Medicine Complementary and Alternative Medicine in the United States. Washington (DC): National Academies Press (US); 2005. 22379647

[16] Zampogna B Papalia R Papalia GF The role of physical activity as conservative treatment for hip and knee osteoarthritis in older people: a systematic review and meta-analysis. J Clin Med 2020;9:1167. 10.3390/jcm9041167 PMC7230847 32325775

[17] Chen B Zhan H Marszalek J Traditional Chinese medications for knee osteoarthritis pain: a meta-analysis of randomized controlled trials. Am J Chin Med 2016;44:677–703. 27222066 10.1142/S0192415X16500373 PMC5553612

[18] Yang M Jiang L Wang Q Traditional Chinese medicine for knee osteoarthritis: an overview of systematic review. PLoS One 2017;12:e0189884. 29267324 10.1371/journal.pone.0189884 PMC5739454

[19] Field T Knee osteoarthritis pain in the elderly can be reduced by massage therapy, yoga and tai chi: a review. Complement Ther Clin Pract 2016;22:87–92. 26850812 10.1016/j.ctcp.2016.01.001

[20] Stein J Banzer W Treating chronic knee pain with acupuncture. JAMA 2015;313:626–7. 10.1001/jama.2014.18505 25668271

[21] Chen R Chen M Su T Heat-sensitive moxibustion in patients with osteoarthritis of the knee: a three-armed multicentre randomised active control trial. Acupunct Med 2015;33:262–9. 25998755 10.1136/acupmed-2014-010740

[22] Lao L Hochberg M Lee DYW Huo-Luo-Xiao-Ling (HLXL)-Dan, a Traditional Chinese Medicine for patients with osteoarthritis of the knee: a multi-site,randomized, double-blind, placebo-controlled phase II clinical trial. Osteoarthr Cartil 2015;23:2102–8. 10.1016/j.joca.2015.06.007 PMC4663117 26099553

[23] Deepeshwar S Tanwar M Kavuri V Effect of yoga based lifestyle intervention on patients with knee osteoarthritis: a randomized controlled trial. Front Psychiatry 2018;9:180. 29867604 10.3389/fpsyt.2018.00180 PMC5952125

[24] Wang C Schmid CH Hibberd PL Tai Chi is effective in treating knee osteoarthritis: a randomized controlled trial. Arthritis Rheum 2009;61:1545–53. 19877092 10.1002/art.24832 PMC3023169

[25] Chen N Wang J Mucelli A Electro-acupuncture is beneficial for knee osteoarthritis: the evidence from meta-analysis of randomized controlled trials. Am J Chin Med 2017;45:965–85. 28659033 10.1142/S0192415X17500513

[26] Choi TY Lee MS Kim JI Moxibustion for the treatment of osteoarthritis: an updated systematic review and meta-analysis. Maturitas 2017;100:33–48. 28539175 10.1016/j.maturitas.2017.03.314

[27] Kan L Zhang J Yang Y The effects of yoga on pain, mobility, and quality of life in patients with knee osteoarthritis: a systematic review. Evid Based Complement Alternat Med 2016;2016:6016532. 27777597 10.1155/2016/6016532 PMC5061981

[28] Lauche R Hunter DJ Adams J Yoga for osteoarthritis: a systematic review and Meta-analysis. Curr Rheumatol Rep 2019;21:47. 31338685 10.1007/s11926-019-0846-5

[29] Lauche R Langhorst J Dobos G A systematic review and meta-analysis of Tai Chi for osteoarthritis of the knee. Complement Ther Med 2013;21:396–406. 23876571 10.1016/j.ctim.2013.06.001

[30] Zeng ZP Liu YB Fang J Effects of Baduanjin exercise for knee osteoarthritis: a systematic review and meta-analysis. Complement Ther Med 2020;48:102279. 31987253 10.1016/j.ctim.2019.102279

[31] Shamseer L Moher D Clarke M Preferred reporting items for systematic review and meta-analysis protocols (PRISMA-P) 2015: elaboration and explanation. BMJ 2015;350:g7647. 25555855 10.1136/bmj.g7647

[32] Gandek B Measurement properties of the Western Ontario and McMaster Universities Osteoarthritis Index: a systematic review. Arthritis Care Res (Hoboken) 2015;67:216–29. 25048451 10.1002/acr.22415

[33] Treacy D Hassett L The short physical performance battery. J Physiother 2018;64:61. 28645532 10.1016/j.jphys.2017.04.002

[34] Mehta SP Fulton A Quach C Measurement properties of the lower extremity functional scale: a systematic review. J Orthop Sports Phys Ther 2016;46:200–16. 26813750 10.2519/jospt.2016.6165

[35] Heller GZ Manuguerra M Chow R How to analyze the visual analogue scale: myths, truths and clinical relevance. Scand J Pain 2016;13:67–75. 28850536 10.1016/j.sjpain.2016.06.012

[36] Mørup-Petersen A Holm PM Holm CE Knee osteoarthritis patients can provide useful estimates of passive knee range of motion: development and validation of the Copenhagen Knee ROM Scale. J Arthroplasty 2018;33:2875–83.e3. [published correction appears in J Arthroplasty 2019 Aug;34(8):1860–1]. 29887360 10.1016/j.arth.2018.05.011

[37] Downs S The Berg Balance Scale. J Physiother 2015;61:46. 25476663 10.1016/j.jphys.2014.10.002

[38] Farah NM Saw Yee T Mohd Rasdi HF Self-reported sleep quality using the Malay Version of the Pittsburgh Sleep Quality Index (PSQI-M) in Malaysian adults. Int J Environ Res Public Health 2019;16:4750. 10.3390/ijerph16234750 PMC6926836 31783607

[39] Contopoulos-Ioannidis DG Karvouni A Kouri I Reporting and interpretation of SF-36 outcomes in randomised trials: systematic review. BMJ 2009;338:a3006. 19139138 10.1136/bmj.a3006 PMC2628302

[40] Higgins JPT Green S Cochrane handbook for systematic reviews of interventions Version 5.1.0 [updated March 2011]. The Cochrane Collaboration 2011.

[41] Van Valkenhoef G Tervonen T Zwinkels T ADDIS: a decision support system for evidence-based medicine. Decis Support Syst 2013;55:459–75.

[42] Guyatt G Oxman AD Akl EA GRADE guidelines: 1. Introduction-GRADE evidence profiles and summary of findings tables. J Clin Epidemiol 2011;64:383–94. 21195583 10.1016/j.jclinepi.2010.04.026

[43] Vaghela N Mishra D Patel J Promoting health and quality of life of patients with osteoarthritis of knee joint through non-pharmacological treatment strategies: a randomized controlled trial. J Educ Health Promot 2020;9:156. 32766341 10.4103/jehp.jehp_39_20 PMC7377148

